# Nucleologenesis in the *Caenorhabditis elegans* Embryo

**DOI:** 10.1371/journal.pone.0040290

**Published:** 2012-07-02

**Authors:** Darina Korčeková, Adriána Gombitová, Ivan Raška, Dušan Cmarko, Christian Lanctôt

**Affiliations:** Institute of Cellular Biology and Pathology, First Faculty of Medicine, Charles University, Prague, Czech Republic; University of Louisville, United States of America

## Abstract

In the *Caenorhabditis elegans* nematode, the oocyte nucleolus disappears prior to fertilization. We have now investigated the re-formation of the nucleolus in the early embryo of this model organism by immunostaining for fibrillarin and DAO-5, a putative NOLC1/Nopp140 homolog involved in ribosome assembly. We find that labeled nucleoli first appear in somatic cells at around the 8-cell stage, at a time when transcription of the embryonic genome begins. Quantitative analysis of radial positioning showed the nucleolus to be localized at the nuclear periphery in a majority of early embryonic nuclei. At the ultrastructural level, the embryonic nucleolus appears to be composed of a relatively homogenous core surrounded by a crescent-shaped granular structure. Prior to embryonic genome activation, fibrillarin and DAO-5 staining is seen in numerous small nucleoplasmic foci. This staining pattern persists in the germline up to the ∼100-cell stage, until the P4 germ cell divides to give rise to the Z2/Z3 primordial germ cells and embryonic transcription is activated in this lineage. In the *ncl-1* mutant, which is characterized by increased transcription of rDNA, DAO-5-labeled nucleoli are already present at the 2-cell stage. Our results suggest a link between the activation of transcription and the initial formation of nucleoli in the *C. elegans* embryo.

## Introduction

The nucleolus is the most prominent compartment in the cell nucleus. It is the site of rRNA synthesis and processing, as well as of biogenesis of the ribosomal subunits [1]. In recent years, the nucleolus has been implicated in a range of other cellular functions, including cell cycle regulation and stress sensing [2]. The nucleolus is a dynamic structure that disassembles when transcription of rDNA ceases and reassembles when transcription resumes (reviewed in [3]). One of the most visible manifestation of such a cycle occurs during mitosis in most metazoans, when the majority of rRNA processing enzymes and ribosome assembly components exit the nucleolar compartment in early prophase only to reassemble into a distinct nucleolus after having transited through cytoplasmic nucleolus-derived foci (NDF) in anaphase and/or prenucleolar bodies (PNB) in telophase/early G1 [4], [5]. In this process, the trigger for disassembly is the phosphorylation/inactivation of factors involved in the initiation of RNA polymerase I-mediated transcription [6]. Conversely, nucleolar re-assembly is linked to the dephosphorylation/re-activation of these same factors toward the end of mitosis.

Another example that highlights the dynamic nature of the nucleolus is to be found in early embryogenesis. Indeed in many species the nucleolus disappears at late stages of oogenesis and spermatogenesis and reappears at various times after fertilization. In the mouse for instance, the initial transition from a transcriptionally-active “nonsurrounded nucleolus” oocyte to a silent “surrounded nucleolus” one is accompanied by a redistribution of the B23/nucleophosmin nucleolar protein from the periphery of the nucleolus-like body to the nucleoplasm [7]. The disappearance of the nucleolus is gradual, culminating with the dissolution of the nucleolus-like body and the degradation of RNA polymerase I upon germinal vesicle breakdown. The reformation of the nucleolus during early embryogenesis has been well studied in *Xenopus laevis*. In this species, nucleolar proteins such as fibrillarin and nucleolin were found to cluster on rDNA upon embryonic genome activation [8]. Earlier in embryogenesis, fibrillarin was detected in numerous dot-like nucleoplasmic structures reminiscent of the mitotic PNBs.

In *Caenorhabditis elegans*, transcription is silenced as oocytes enter the diakinetic stage of meiosis, as shown by the lack of immunostaining using antibodies against either the initiating or the elongating forms of RNA polymerase II [9]. Presumably this inhibition extends to RNA polymerase I, since the nucleolus disappears at about the same time. To date, very few studies have addressed the issue of nucleolar reformation during early embryogenesis of *C. elegans*. Sasano *et al.* looked at the distribution of U3 snoRNA and fibrillarin during early development and found co-localization of these nucleolar components in nuclear foci starting at the 4–8-cell stage [10]. Also, it has been noted that GFP fusions of nucleolar proteins involved in rRNA processing (RBD-1) or ribosome biogenesis (nucleostemin) under the control of their own promoters were not expressed before morphogenesis or the ∼18-cell stage, respectively [11], [12]. In the present work, we have extended these studies and carried out a systematic analysis of nucleologenesis during *C. elegans* embryogenesis. To do so, we have relied on the immunolabeling of both fibrillarin and DAO-5, the *C. elegans* homologue of NOLC1/Nopp140. Nopp140 is a well-characterized nucleolar protein which localizes to the dense fibrillar component in the nucleolus [13]. Transcription and initial processing of rRNA takes place in this compartment and/or at its interface with the inner fibrillar center [14]–[16]. Unlike components of the RNA polymerase I machinery, Nopp140 does not remain associated with the nucleolar-organizing regions during mitosis [17], an observation which suggests that the protein is involved in pre-rRNA processing rather than transcription. Using fibrillarin and DAO-5/Nopp140 as markers, we found that distinct nucleoli appear at around the time of embryonic genome activation in somatic and germ cells of the *C. elegans* embryo.

## Materials and Methods

### *C. elegans* Strains and Antibodies

The *Caenorhabditis elegans* strain N2 var Bristol was used as wild type. The *ncl-1(e1865)* strain was provided by the Caenorhabditis Genetics Center. Worms were maintained according to standard protocols [18]. The mouse monoclonal antibody (clone 5E9) against an His_6_-tagged fusion protein to the last 220 amino acids of DAO-5 (WP:CE08376, Wormbase release WS225) was developed by M. L. Nonet and collaborators, who validated it by Western blotting and immunofluorescence on adult gonads [19]. It was obtained from the Developmental Studies Hybridoma Bank (University of Iowa, USA). The rabbit monoclonal antibody against a synthetic peptide surrounding Thr298 of human fibrillarin was obtained from Cell Signaling Technology (clone C13C3, cat. no. 2639). The mouse monoclonal antibody against fibrillarin was obtained from Abcam (clone 38F3, cat no. ab4566). The rabbit polyclonal antibody against PGL-1 was a kind gift of Dr. Susan Strome [20]. HeLa cells were obtained from the ATCC (no. CCL-2). Biotin- or fluorophore-conjugated highly cross-adsorbed secondary antibodies were purchased from Jackson ImmunoResearch.

### Immunofluorescence Labeling

Our immunostaining protocol was based on published procedures [21]. Briefly, young gravid worms were dissolved in bleaching solution (0.5M NaOH/0.8% sodium hypochlorite) for 5–8 minutes to release embryos. After washing, embryos were deposited on poly-L-lysine treated slides and slightly compressed under a coverslip. The eggshell was freeze-cracked after incubation at −80°C for at least 60 minutes. Embryos were immediately fixed in cold methanol for 2 minutes at −20°C. Slides were then transferred either to cold 4% formaldehyde (DAO-5 staining) or to −20°C acetone (fibrillarin staining). Samples were fixed for 10 minutes either at −20°C for acetone or at room temperature for formaldehyde. In the case of acetone fixation, samples were then rehydrated through 70%, 50% and 30% acetone in PBS. For co-localization experiments, fixation was done in acetone. After fixation, samples were incubated 5 minutes in PBS/0.02% Tween 20 (PBST) and then 2 hours at room temperature in dilutions of primary antibodies in either PBST/0.5% bovine serum albumin (BSA)/0.5% dry milk (1/200 for anti-DAO-5, 1/5000 for anti-PGL-1), PBS/1% BSA/1% normal goat serum (1/40 for rabbit anti-fibrillarin) or PBST/1% BSA/10% normal goat serum (1/500 for mouse anti-fibrillarin). Incubations with secondary antibodies (1/400 dilution) were performed for 1 hour at room temperature. DNA was counterstained with 4′,6-diamidino-2-phenylindole (DAPI) at 1 µg/ml. Samples were mounted in Vectashield (Vector Labs, USA).

### Immuno-DNA FISH

A 390 bp fragment encompassing nt 172–562 upstream of the 18S rDNA sequence (*rrn-1.1*) was amplified from *C. elegans* genomic DNA using forward primer TTGTGCAAGCGGCCGAGGTC and reverse primer AGACTCAAGCGCCTCGACGC. To obtain a labeled probe, Cy3-dUTP was included in the PCR reaction mix at a final concentration of 65 µM (ratio Cy3-dUTP/dTTP of 1∶2). Immunolabeling with the DAO-5 antibody was performed as described above. After incubation with a 1/200 dilution of biotinylated anti-mouse antibody, the immunocomplexes were fixed *in situ* for 10 minutes at room temperature with 2% formaldehyde in PBS. Slides were then successively treated as follows: 0.1 N HCl, 2 minutes; 50 µg/ml RNAse A in 2X SSC, 45 minutes at 37°C; 2XSSC/50% formamide, 2 hours. Samples were pre-hybridized overnight at 37°C in 2XSSC/50% formamide/10% dextran sulfate supplemented with 1 ng/µl of Cy3-labeled probe. Slides were placed on a hot plate at 76°C for 5 minutes to simultaneously denature probe and target DNA. Hybridization was carried out for 3 days at 37°C. Slides were washed 3 times at 37°C in 2XSSC and twice at 55°C in 0.2XSSC. Biotinylated immunocomplexes were detected using a 1/400 dilution of Avidin-Alexa488 (Invitrogen).

### RNA FISH

A 412 bp fragment comprising almost all of the first internal transcribed spacer (ITS1) of the *C. elegans* pre-rRNA was amplified from genomic DNA using forward primer CTGCAGCTGGATCATCGCCG and reverse primer CAAATCACCGCATGTCCGTG. To obtain a labeled probe, Atto647N-dUTP was included in the PCR reaction mix at a final concentration of 65 µM (ratio Atto647N-dUTP/dTTP of 1∶2). Embryos were obtained and fixed in formaldehyde as described above. Samples were permeabilized with 0.5% Triton X-100 in PBS 1X (5 minutes) and equilibrated in 2XSSC/10% formamide for 15 minutes at room temperature. Negative control slides were treated with 50 µg/ml RNAse A in 2XSSC for 30 minutes at 37°C. Hybridization was carried out with 2 ng/µl of denatured Atto647N-labeled probe in 2XSSC/10% formamide/10% dextran sulfate (∼16–18 hours at 42°C). Slides were rinsed in 2XSSC, washed twice at 42°C in 10% formamide/1X SSC and, after DAPI staining, mounted in Vectashield.

### Immunoelectron Microscopy

Embryos were collected as described above. After three washes in M9, embryos were resuspended in 50 µl of M9 supplemented with 20% BSA. A volume of 0.7 µl (containing 25–50 embryos) was frozen under high pressure using the Leica EM PACT2 instrument. Samples were then cryo-substituted in acetone and embedded in Lowicryl HM20 (EMS Inc., USA) at low temperature in a Leica EM AFS2. The cryo-substitution solution contained 0.1% (w/v) uranyl acetate. Polymerization was initiated under UV light at −50°C and proceeded with gradual warming to room temperature. Ultrathin sections (60 nm) were deposited on formvar/carbon-coated grids. After blocking in a 1% normal goat serum solution in PBS for 10 minutes, samples were immunolabeled overnight at 4°C with a 1/5 dilution of the DAO-5 hybridoma medium in PBS/0.025% Tween 20/0.1% BSA. Blocking was performed as before prior to incubation with goat anti-mouse antibody coupled to 12 nm gold particles (1/10 dilution in PBS, Jackson ImmunoResearch) for 45 minutes at room temperature. After washes in PBS and distilled water, samples were counterstained with 4% uranyl acetate for 10 minutes and lead citrate for 4 minutes. Observation was carried out on a Zeiss 900 electron microscope.

### Image Acquisition, Analysis and Quantification

Images were acquired on a Leica SP5 scanning confocal microscope with a HCX Plan Apochromat lambda blue 63X oil objective (numerical aperture of 1.4). Image processing was performed using ImageJ v1.45i (http://imagej.nih.gov/ij/). Peripheral localization was assessed using the eADS (enhanced Absolute 3D Distances to Surface) program. This custom-built MATLab program, kindly provided by Prof. Thomas Cremer, determines the shortest distance between every voxel belonging to a thresholded signal and the closest border of the nucleus, as defined by a thresholded mask of the DNA counterstain [22].

## Results

### Immunolocalization of Nucleolar Markers during *C. elegans* Development

*Dao-5* was first identified by differential display PCR as a gene that is overexpressed in long-lived *daf-2* mutants [23]. The gene is predicted to give rise to two isoforms, both of which encode *C. elegans* homologs of human Nopp140. The similarity between hNopp140 (699 amino acids) and the short 696-residues isoform of *C. elegans* DAO-5 is 55% (Figure S1). Like its mammalian counterparts, DAO-5 comprises alternating stretches of serine and acidic residues in its central region and a SRP40 domain at its C-terminus. In the present study, DAO-5 was chosen as one of the non ribosomal nucleolar markers used to follow nucleologenesis during development of *C. elegans*, the other being the well-characterized fibrillarin protein (FIB-1). Immunofluorescence experiments to detect DAO-5 were performed using a newly-available monoclonal antibody raised against the last 220 amino acids of the *C. elegans* protein [19]. Despite the fact that the epitope encompasses the conserved SRP40 domain, the DAO-5 antibody did not cross-react with human Nopp140 as judged by the absence of nucleolar signal in HeLa cells (Figure S2).

**Figure 1:**
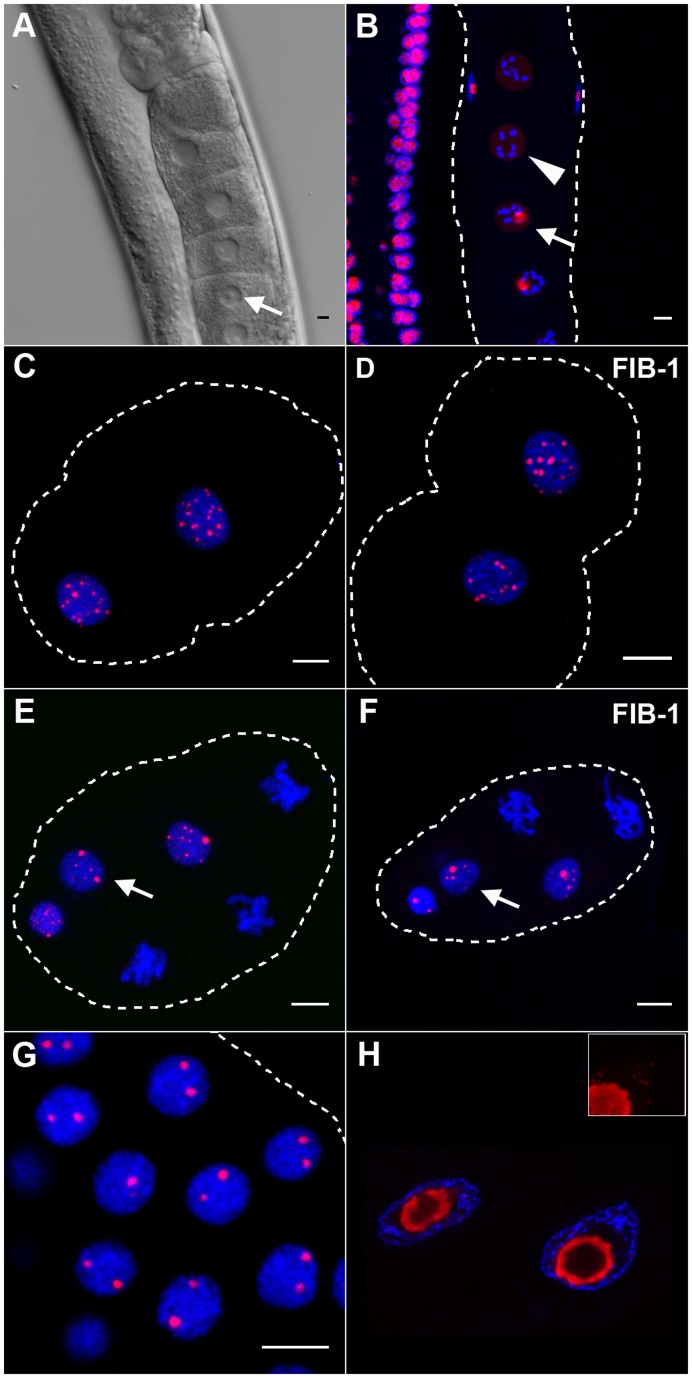
Immunolocalization of nucleolar markers in embryonic and adult nuclei. A, B) In the adult gonad, prominent nucleoli are easily detected in all nuclei (arrows), except in those of the most mature oocytes. Accordingly, only a faint nucleoplasmic DAO-5 signal (in red) can be detected in these late oocytes (arrowhead). C, D) At the 2-cell stage, the DAO-5 and FIB-1 antibodies label numerous dots of varying size and intensity throughout the nucleoplasm. E,F) The first appearance of distinct nucleoli (arrows) occurs at the 6- to 8-cell stage. Shown here are projections of parts of 8-cell embryos. G) At later stages, all somatic cells display one, or in most cases two DAO-5-labeled nucleoli. Shown here is a 2.5 µm slice from a 45-cell embryo. H) On this single optical section of adult intestinal nuclei, DAO-5 is clearly found in ring-like structures in a DNA-poor region. On a projection of all optical sections, numerous dot-like DAO-5-positive structures are seen throughout the nucleoplasm (inset). In this and other figures, the contour of the labeled embryos is dotted and DNA is counterstained with DAPI (in blue). Bars: 5 µm.

In *C. elegans*, transcription shuts down shortly prior to fertilization during the diakinesis stage of meiotic prophase [9]. Accordingly, the nucleolus disappears completely in the most mature oocytes (Figure 1A–B). In the rest of the gonad, every germline nucleus displays a single large DAO-5-positive nucleolus. In the 2-cell stage embryo, the DAO-5 signal consists of 15–25 small dots of varying intensity distributed throughout each nucleus (Figure 1C). A similar staining pattern was observed for FIB-1 (Figure 1D). Nucleolus-like structures were never observed at this early stage. The first appearance of a nucleolar structure occurs at the 6- to 8-cell stage, when larger spherical bodies (∼0.5 µm in diameter) are immunodetected amidst the dot-like signals (Figure 1E–F). Shortly after, the number of small dot-like signals that are detected decreases drastically (but some persist until later stages) and, except for the germline cell (see below), all embryonic nuclei then harbor one or, in the vast majority, two distinct DAO-5 or FIB-1 signals of equal size and intensity (Figure 1G and Figure S3). This pattern remains broadly similar throughout embryogenesis, with the notable exception that intestinal nuclei often contain a single large DAO-5/FIB-1 positive nucleolus at later stages (not shown). It should be noted that at all embryonic stages examined, a substantial pool of diffuse nucleoplasmic DAO-5 was also detected (Figure S4). Control experiments performed by omitting the primary antibody incubation or the DAPI counterstain showed that this nucleoplasmic signal was neither background nor the result of bleed-through of the strong DAPI signal in the detection channel. In the adult, the localization of DAO-5 was examined in more detail in the polyploid intestinal nuclei, which contain a large centrally-located nucleolus. In these nucleoli, the DAO-5 signal describes a hollow sphere that surrounds a DNA-poor core. The signal is seen as a ring on single optical sections (Figure 1H). In addition to being present in this portion of the nucleolus, DAO-5 is found in numerous small punctate structures throughout the nucleoplasm of intestinal nuclei (Figure 1H, inset).

**Figure 2:**
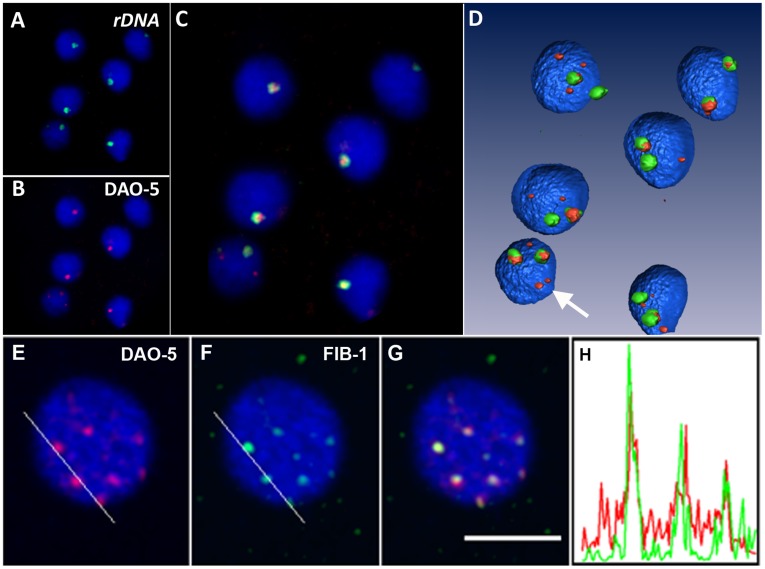
Co-localization of DAO-5 with rDNA or fibrillarin. Immunolabeling and DNA FISH were performed sequentially on the same sample. Single optical section of part of a 30-cell embryo showing the rDNA signal (A), the DAO-5 signal (B) and the merged image (C). Shown in D is a 3D reconstruction of part of the sample. Note the presence of occasional DAO-5-positive bodies in the nucleoplasm of embryonic nuclei (arrow). E-H) Immunostaining was performed for DAO-5 and FIB-1 in early embryos. Single optical section (thickness of 800 nm) showing the DAO-5 signal (in red, E) in the nucleus of a 2-cell embryo. FIB-1 signal (in green, F) on the same optical section. Overlay of the DAO-5 and FIB-1 signals (G, scale bar: 5 µm). Intensity profiles (H) along the lines shown in E (DAO-5, red) and in F (FIB-1, green).

To ascertain that the pair of DAO-5 signals detected during embryogenesis corresponds to *bona fide* nucleoli, their localization relative to rDNA was assessed using immuno-DNA FISH. The *C. elegans* genome contains a single rDNA cluster of 100–150 repeats at the end of chromosome I [24]. As expected, a 390 bp probe derived from the region upstream of the 18S rDNA sequence (nt −562 to −172) labeled two foci per nucleus and these were invariably the main sites of DAO-5 accumulation (Figure 2A–C). To ascertain that the FISH signal corresponded to rDNA and not to rRNA, hybridization was carried out without prior denaturation of the sample; no signal was detected. Co-localization is clearly seen in a 3D reconstruction of part of a 30-cell embryo, as is the occasional presence of smaller dot-like DAO-5-positive structures in the nucleoplasm (Figure 2D). Interestingly, the rDNA and DAO-5 signals did not co-localize perfectly and the shape of the rDNA signal was found to be somewhat more irregular than that of the DAO-5 one. At early stages, the FISH signal did not co-localize with DAO-5-positive dot-like structures that are detected before the appearance of distinct nucleoli (not shown). To better characterize the molecular composition of these dot-like structures, we performed co-localization experiments with FIB-1. Results show that the majority of early DAO-5 positive nucleoplasmic foci also contained FIB-1 (Figure 2E–H and Figure S5A–C). However, individual nucleoplasmic foci labeled with only one of the markers were occasionally observed (Figure S5D–F). At later stages, co-localization of DAO-5 and FIB-1 was complete (Figure S5G–I).

**Figure 3:**
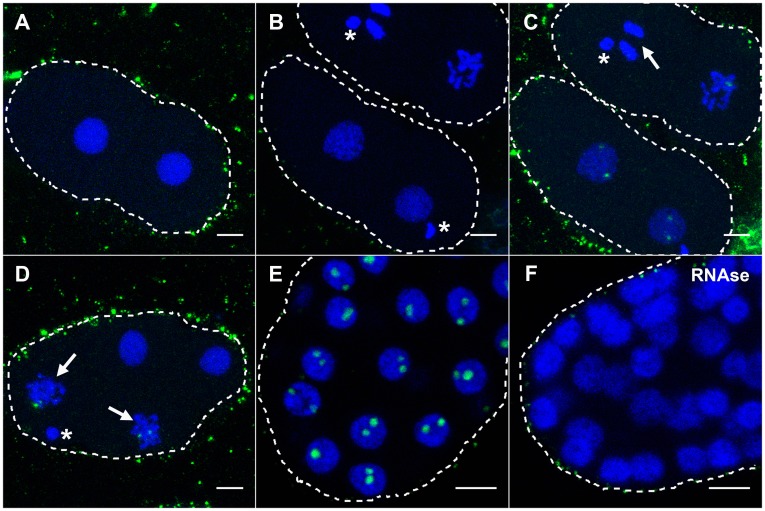
Detection of pre-rRNA molecules in early embryos. RNA FISH was performed using a probe against ITS1 (signal in green). DNA is counterstained with DAPI (blue). Asterisks indicate the remnants of the polar bodies. A dotted-like distribution of RNA FISH signals was never observed. Images A, C and D were overexposed to reveal weak signals (also revealed upon overexposure are probe aggregates non specifically bound to the eggshell of the embryo). A) A 2-cell embryo with both cells in S-phase shows no signal, even upon overexposure. B, C) Two-cell embryos with cells at different stages of mitosis display weak signals only upon overexposure (panel C). Note the absence of signal in anaphase (arrow in C). D) The difference between nuclei in S-phase (negative) and in prophase (arrows, positive) is clearly seen in this 4-cell embryo. E) At later timepoints (here a ∼80-cell embryo), robust signals are detected in the nucleoli (compare B and E, same exposition). F) No signal is detected after RNAse treatment.

The DAO-5/FIB-1 positive nucleoplasmic foci that we observe in early *C. elegans* embryos are reminiscent of the prenucleolar bodies that are detected in the last stages of mitosis and that coalesce on rDNA in early G1 [25]. In addition to processing components (e.g. fibrillarin, U3 snoRNA), it has been reported that these PNBs contain unprocessed or partially processed rRNA [26]. In order to determine whether the DAO-5/FIB-1 nucleoplasmic foci share this property of PNBs, we performed RNA FISH on early embryos using a probe against the first internal transcribed spacer (ITS1) of the pre-rRNA molecule. We never observed a dotted-like distribution of RNA FISH signals. On the contrary, in all nuclei in which pre-rRNAs were detected, it was in two distinct spots reminiscent of “nucleolar” localization (Figure 3). RNA FISH signals varied greatly in intensity between stages and within a single embryo. In particular, only prophase nuclei displayed signals in the earliest embryos (Figure 3A–D), and these were much weaker than at later time points (Figure 3E). RNAse treatment prior to hybridization led to a complete disappearance of the signal, indicating that it arose from hybridization to RNA (Figure 3F). Taken together, our results show that the DAO-5/FIB-1 positive nucleoplasmic foci found in the earliest embryos do not contain significant amount of unprocessed or partially processed rRNA.

**Figure 4:**
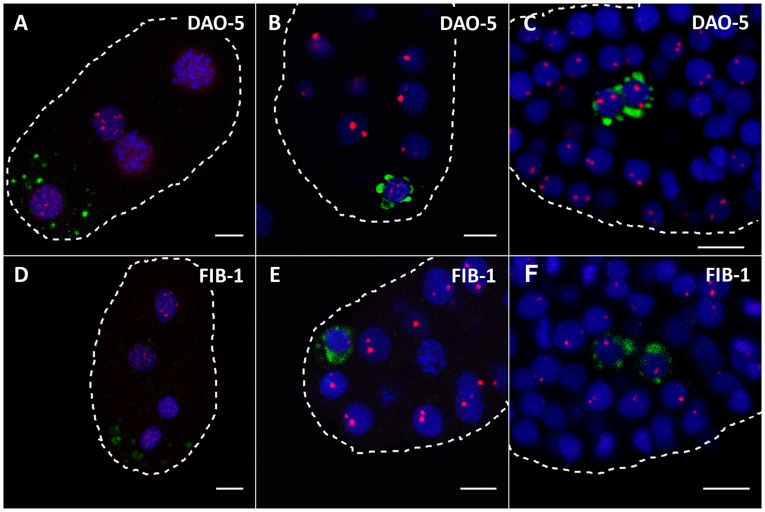
Distinct localization of nucleolar markers in the early germline. In each image, DAO-5 (A–C) or FIB-1 (D–F) is in red; PGL-1, a marker of the germline, is in green; DNA is in blue. A, D) In the 4-cell embryo, the distribution of nucleolar markers is dotted in both somatic and germ cells. B, E) At later stages (here around 25-cell), the distribution of nucleolar markers remains dotted only in the unique germline precursor. C, F) In yet older embryos, which contain two germ cell precurors (Z2 and Z3), nucleolar markers are found in distinct nucleoli in each of these cells. Note that all images are maximal projections centered on the germ cell nuclei and that, as a consequence, not all somatic nuclei are complete. Bars: 5 µm.

As mentioned above, we repeatedly observed in our analysis of nucleolar markers in early embryos that a single nucleus failed to display the staining pattern found in the other cells. From its small size and peripheral position, we suspected that this was the germline nucleus. In order to ascertain the identity of this cell, we performed immunolabeling with an antibody raised against PGL-1, a component of germ granules, i.e. ribonucleoprotein complexes that segregate with germline cells in the early embryo [27]. Comparison of the distribution of DAO-5 and FIB-1 in the germline and in the soma during embryogenesis revealed that the staining pattern for nucleolar markers remains dotted-like much longer in the former (Figure 4). Indeed, in 24 out of 27 embryos between the 8- and ∼100-cell stage DAO-5 was found in numerous small dot-like structures in the single germline cell that is present at these stages, similar to what is observed in all nuclei at the earliest timepoints, e.g. in the 4-cell embryo (Figure 4A–B). In the other 3 embryos, the germline DAO-5 staining pattern consisted of fewer and weaker dot-like structures, two of which appeared slightly more intense than the rest. Fibrillarin showed a similar pattern: in 31 out of the 36 embryos that were analyzed, the FIB-1 signal in the germline was either dot-like (21/31) or weak and diffuse throughout the nucleoplasm (10/31) (Figure 4D–E). We believe that the weak FIB-1 signals detected in a high proportion of germline nuclei is likely due to the poor fixation properties of acetone, which had to be used in order for the anti-fibrillarin antibody to work. Indeed, we also observed weaker and more diffuse signals when immunostaining for DAO-5 was carried out after fixation with acetone instead of formaldehyde. In the other embryos immunostained for fibrillarin (5/31), the single germline nuclei contained fewer FIB-1-positive dot-like structures and in each case two of these appeared slightly more intense, as was observed for DAO-5 in 3 out of 27 germline nuclei. Notwithstanding the observation of minor differences in staining patterns, it should be emphasized that in all embryos that were included in the preceding analysis (n = 63), the intensity and distribution of nucleolar markers in the germline nuclei were clearly distinct from those in the neighboring somatic cells, which all showed pairs of clear and robust DAO-5 and FIB-1 signals. It is only once the P4 cell has divided into the Z2 and Z3 primordial germ cells that DAO-5 and FIB-1-positive nucleoli are detected in these cells (Figure 4C, F). The latter observation was made in 40 out of 40 such primordial germ cells (n = 20 embryos).

### Immunolocalization of DAO-5 in Ncl-1 Mutants and during Mitosis

**Figure 5:**
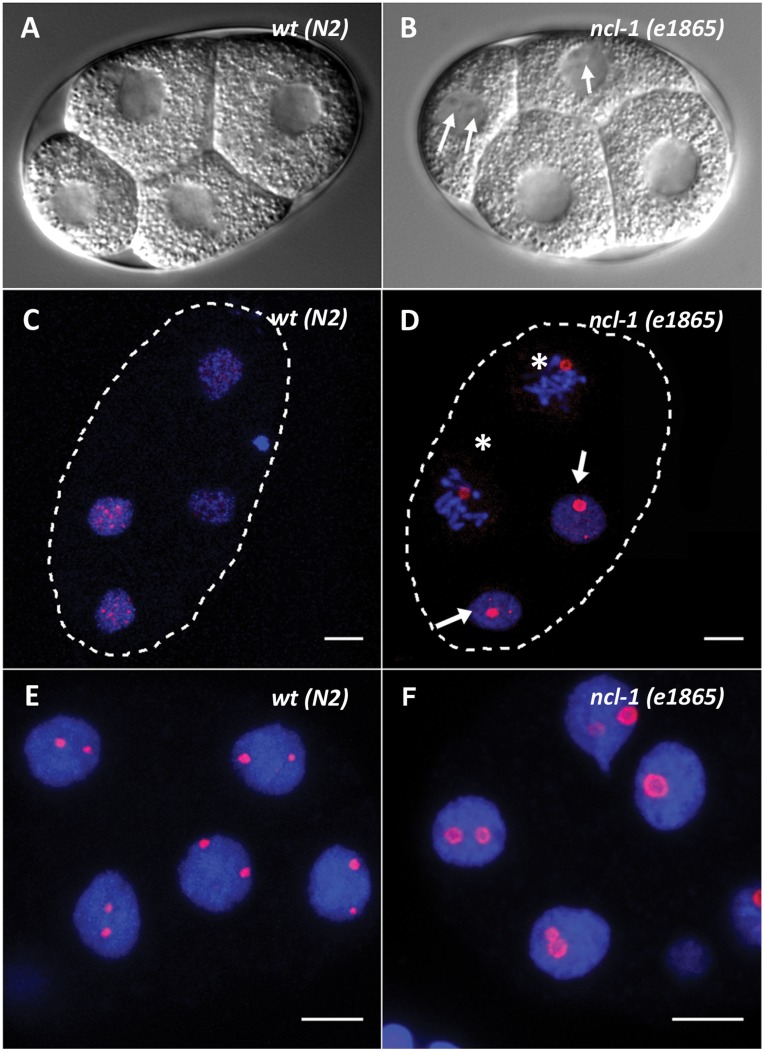
Immunolocalization of DAO-5 in *ncl-1* mutants. In *ncl-1* mutants, well-formed DAO-5-positive nucleoli can be detected at the 4-cell stage (B and D arrows), which is never the case in wildtype embryos (A and C). Note that DAO-5-positive structures persist into prophase in early *ncl-1* mutants (asterisks in D). At later stages (here 14-cell), nucleoli are bigger in *ncl-1* mutants (F) than in wildtype (E). DAO-5 labeling describes a ring-like structure in the mutant strain. Bars: 5 µm.

In *C. elegans*, mutations in the *ncl-1* gene lead to enlargement of the nucleoli in nearly all cells of the adult worm [28]. The *ncl-1* gene encodes a zinc finger protein that is thought to repress ribosome synthesis and cell growth [29]. It was therefore of interest to compare the localization of DAO-5 in wild type and *ncl-1* embryos. As previously reported, we found that well-defined nucleoli can be seen in the 4-cell *ncl-1* embryo by differential interference contrast microscopy, which was never the case in wild type 4-cell embryos (Figure 5A–B). These nucleoli are immunostained by the DAO-5 antibody (Figure 5D). DAO-5-positive nucleoli were also observed in 2-cell embryos (not shown). As in wild type nuclei at this early stage, the DAO-5 antibody also labels numerous small nucleoplasmic punctate structures in *ncl-1* mutants, albeit less intensely (Figure 5C–D). At later stages (∼15-cell), *ncl-1* mutants display enlarged nucleoli of 1.17±0.13 µm in diameter (n = 14). By comparison, the diameter of the nucleolus at this stage is 0.74±0.16 µm in wild type embryos (n = 15). On optical sections, the DAO-5 signal clearly appears as a ring-like structure in *ncl-1* mutants (Figure 5E–F).

**Figure 6:**
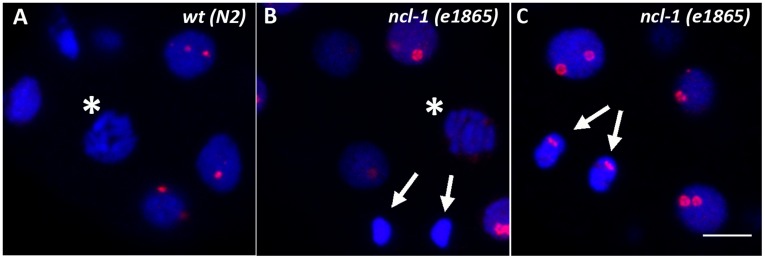
Immunolocalization of DAO-5 during mitosis. A) DAO-5 is not detected in mitotic cells of wildtype embryos (asterisk). B) In *ncl-1* mutants, weak and diffuse nucleoplasmic labeling is observed from prophase (asterisk) to telophase (arrows). Putative remnants of DAO-5-positive nucleoli can be detected in prophase. C) DAO-5 reassociates with forming nucleoli in late telophase/early G1. Bars: 5 µm.

It is generally assumed that the behavior of individual nucleolar proteins during mitosis varies depending on their functions. Factors involved in the transcription of rDNA (e.g. RNA polymerase I, UBF) have been shown to remain associated with the nucleolar-organizing regions on the chromosomes throughout mitosis whereas factors involved in rRNA processing or ribosome biogenesis redistribute to the periphery of mitotic chromosomes (e.g. fibrillarin, protein B23) or to the nucleoplasm (e.g. ribosomal protein S6) [25]. In mammalian cells, the Nopp140 protein was found to disperse in the nucleoplasm in early prophase and to re-associate with reforming nucleoli only late in telophase without transiting through prenucleolar bodies [5]. The same is observed for DAO-5 in early *C. elegans* embryonic nuclei (e.g. 4-cell embryo on Figure 5C shows nucleoplasmic signal in prophase cells). In the mitotic cells of later embryos (∼20-cell stage and beyond), the DAO-5 signal could not be reliably detected, presumably because it is too diffuse in the nucleoplasm at these timepoints (Figure 6A). In *ncl-1* mutants however, a diffuse DAO-5 signal could be detected throughout mitosis (Figure 6B). Interestingly, DAO-5 positive chromosome-associated structures persist until prophase in these embryos (see Figure 5D and Figure 6B). In late telophase, DAO-5 relocalizes to the reforming nucleus and accumulates in nascent nucleoli (Figure 6C). No perichromosomal labeling was ever detected.

### Radial Distribution of DAO-5 in Embryonic Nuclei

**Figure 7:**
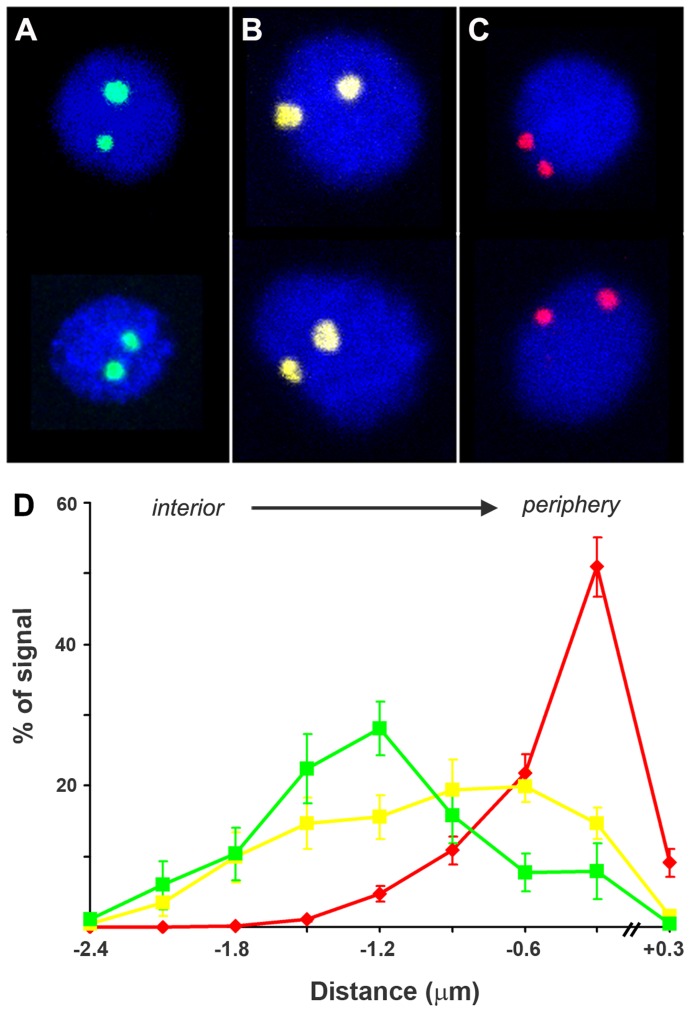
Radial positioning of DAO-5 in embryonic nuclei. The distance between each DAO-5 positive voxel and the nearest nuclear border was measured for 37 embryonic nuclei. Three patterns were observed. Representative nuclei are shown. A) Both nucleoli in the interior of the nucleus (n = 5, 13%, nucleoli pseudo-colored in green). B) One nucleolus at the nuclear periphery and the other in the interior (n = 7, 19%, nucleoli pseudo-colored in yellow). C) Both nucleoli at the periphery (n = 25, 68%, nucleoli pseudo-colored in red). D) For each pattern, mean distances (± standard deviation of the mean) were plotted in bins of 0.3 µm using the color scheme described above. The nuclear periphery is arbitrarily set on the right-hand side of the graph.

We noticed in the course of this work that the DAO-5 signals were often found at the nuclear periphery in the developing *C. elegans* embryo. To quantify this observation, the distribution of DAO-5 was assessed using an algorithm that measures the shortest distance between each signal voxel and the border of the nucleus as defined by the DAPI counterstain (Figure 7). The analysis was carried out on nuclei of embryos between the 15- and the 28-cell stage (n = 37, average nuclear diameter of 5.3±0.5 µm). Results confirmed that in the majority of nuclei (25/37, 68%), both nucleoli were found at the nuclear periphery (83±17% of DAO-5 positive voxels within 600 nm of the nuclear border). In 7 out of 37 nuclei (19%), one of the nucleolus was at the periphery while the other was in the nuclear interior. Finally, 5 nuclei (13%) displayed both nucleoli in the nuclear interior (only 16±15% of DAO-5 positive voxels within 600 nm of the nuclear border). When comparing “periphery” and “interior” patterns, the difference in radial distribution of DAO-5 was highly significant (Mann-Whitney test, *p*<0.0001).

### Ultrastructural Localization of DAO-5

**Figure 8:**
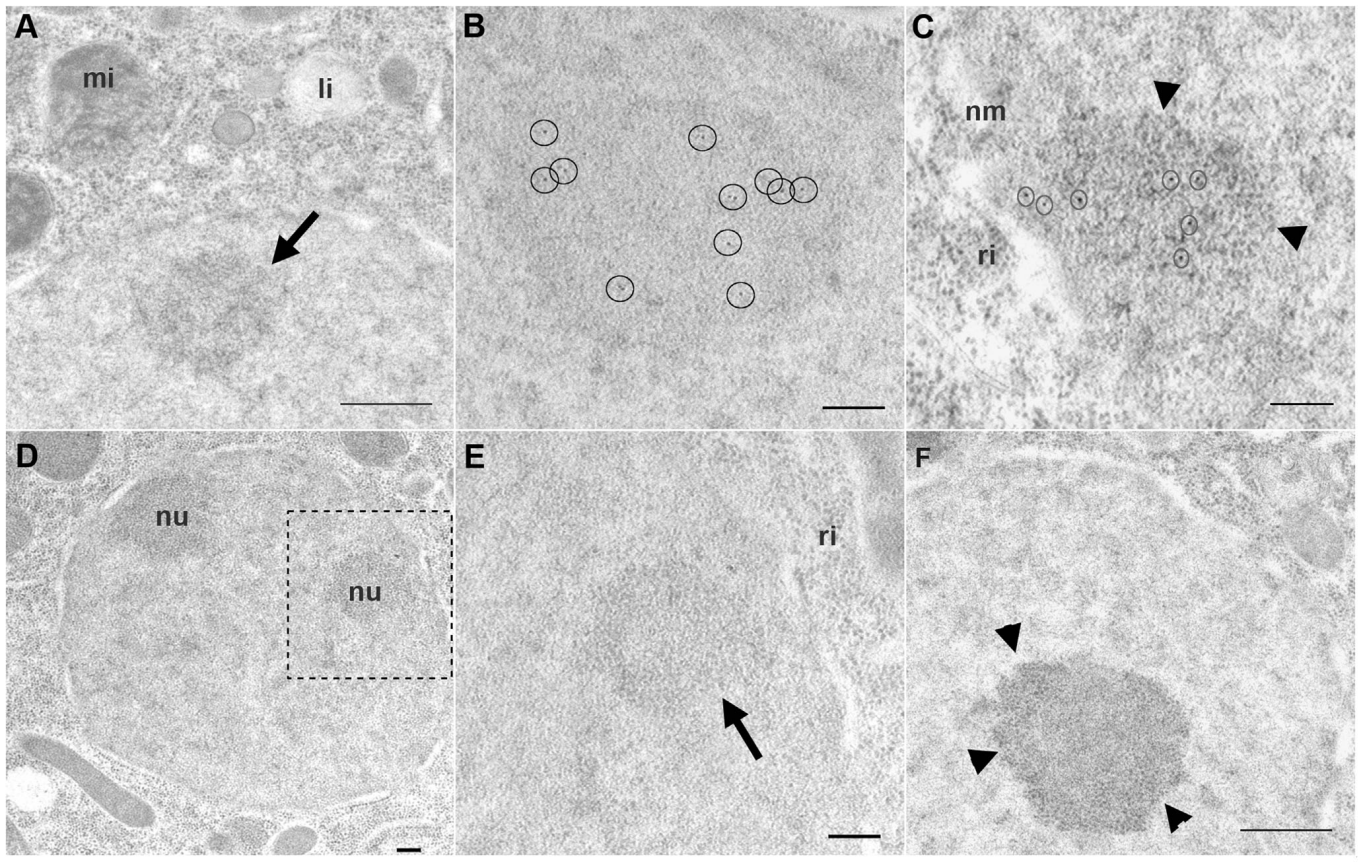
Ultrastructural immunolocalization of DAO-5 in high-pressure frozen and cryo-substituted embryos. A) In early embryos (∼10–20-cell stage), the nucleolus (arrow) appears as an electron-dense structure with no obvious internal organization. B) Post-embedding immunolabeling of DAO-5 confirms that the protein is associated with the nucleolus. C) In slightly later embryos (∼30–40-cell stage), initial signs of granular structures (arrowheads) are found at the periphery of the nucleolus. The DAO-5 signal appears to be excluded from this region. D) A section showing two granular nucleoli in the nucleus of a ∼30–40-cell stage. E) Higher magnification of the region boxed in D. The arrow points to the nucleolus. F) In yet older embryos (∼60–80-cell stage), the segregation of granular structures (arrowheads) at the periphery of the nucleolus is more pronounced. li, lipid droplet; mi, mitochondria; nm, nuclear membrane; ri, ribosomes. Bars: A, D and F, 500 nm; B, C and E, 200 nm.

In order to localize DAO-5 at the ultrastructural level, immunogold-labeling was performed on ultrathin sections of embryos. Samples were frozen at high pressure and cryo-embedded in Lowicryl resin, conditions which afforded excellent preservation of ultrastructure and antigenicity. In rapidly-frozen *C. elegans* embryos (∼10–20 cells), the nucleolus appears as a mainly electron-dense structure with no obvious sign of internal structural differentiation (Figure 8A). Post-embedding immunolabeling confirmed the localization of DAO-5 in the nucleolus (Figure 8B). In slightly older embryo (∼30–40 cells), initial signs of granular structures were often seen at the periphery of the nucleolus (Figure 8C–E). The DAO-5 signal (between 5 and 12 gold particles per nucleolar section) appeared to be excluded from this region (Figure 8C). A segregation of granular structures at the periphery of the nucleolus could often be clearly observed in older embryos (∼60 cells, Figure 8F). While this observation does suggest some form of internal nucleolar organization, the clear compartmentalization that is typical of the mammalian nucleolus could not be observed in high-pressure frozen and cryo-embedded *C. elegans* samples. At least in the case of the innermost compartment, the reason for this difference can be ascribed to the fixation method. Indeed, in the chemically-fixed *C. elegans* nucleolus, fibrillar center-like electron-lucid zones could be easily observed in the nucleolus (Method S1 and Figure S6). It remains to be determined whether a dense fibrillar component (DFC) can be reliably identified in such samples. It is worth noting, however, that a bipartite structure lacking the DFC has been suggested to be a nucleolar feature of eukaryotes which have compact rDNA arrays [30]. With an intergenic region of only ∼940 base pairs in length and a rDNA transcription unit of ∼7200 base pairs, *C. elegans* would undoubtedly qualify as such an organism.

## Discussion

In this work, we have used antibodies directed against fibrillarin (FIB-1) and DAO-5, the *C. elegans* homologue of Nopp140, to investigate the process of nucleolar reformation during embryogenesis of this model organism. The choice of these markers was dictated foremost by the fact that they have been used previously to study nucleologenesis in the early embryo of other model organisms [8], [31], [32], thereby allowing comparisons to be made. Our main finding is that nucleoli appear at around the 6 to 8-cell stage in the somatic nuclei of the *C. elegans* embryo. Prior to that, nucleolar markers are found in nucleoplasmic foci of various sizes and intensities, the precise identity of which remains to be determined. One possibility is that these foci are akin to the PNBs that have been proposed to represent intermediate structures during reassembly of the nucleolus at the end of mitosis [25]. Our finding that these structures contain both DAO-5 and FIB-1 is in agreement with this possibility. On the other hand, our failure to detect pre-rRNAs in the early DAO-5/FIB-1 nucleoplasmic foci is at odds with results showing the presence of unprocessed or partially processed rRNA molecules in the PNBs [26], [33]. Another possibility is that the foci are Cajal bodies, which indeed have been shown in cultured cells to contain Nopp140 and fibrillarin [17], [34] and to be a transit point for Nopp140 on its way to the nucleolus [35]. In the early mouse embryo, Nopp140 co-localizes with coilin, a marker of the Cajal body, in fine punctate structures in the nucleoplasm [31], [36]. Interestingly, a role for Cajal bodies during nucleologenesis has been proposed in the early mouse embryo as putative assembly sites of RNA polymerase I transcription complexes [32]. The comparison with other species, however, can be misleading. Indeed, in the early mouse embryo Nopp140 is found not only in discrete nucleoplasmic foci (as we now report for DAO-5 and FIB-1 in the early *C. elegans* embryo), but also in the cortical region of the prominent “nucleolus-like bodies”, a structure which is thought to act as a scaffold for nucleolar assembly in the mammalian embryo [37], [38], but which is conspicuously absent from the *C. elegans* embryo. To our knowledge, neither Cajal bodies nor an ortholog of the coilin protein have yet been identified in *C. elegans*. However, bioinformatic analysis did reveal that a canonical H/ACA snoRNA found in the *C. elegans* genome is partially homologous to the U85 and U89 Cajal body-specific RNA [39]. Attemps to detect this putative scaRNA by RNA FISH in early *C. elegans* embryos were not successful (data not shown). Hence, the identity and function of the foci labeled by nucleolar markers and dispersed throughout the nucleoplasm in early *C. elegans* blastomeres remain to be determined. The same applies to the few extra-nucleolar DAO-5 foci that are often detected at later stages (e.g. Figure 2D).

Starting from the 6–8-cell stage, a striking redistribution of nucleolar markers occurs: from this point on, each somatic embryonic nucleus harbors two distinct DAO-5 foci that co-localize with rDNA. The appearance of distinct nucleoli in the *C. elegans* embryo seems to coincide with embryonic genome activation (EGA), which in this organism also takes place at around the 8-cell stage [40]. Recent work using a GFP-Nopp140 chimera to image nucleolar formation in living *D. melanogaster* blastomeres found that the initial aggregation of this marker coincided with EGA in this species as well [41]. However, in this case, the GFP-Nopp140 protein was found to be diffusely distributed throughout the nucleus with no signs of clustering prior to EGA, something which we have never observed in the case of *C. elegans* DAO-5. In addition to the temporal coincidence between aggregation of nucleolar markers and EGA, other results indicated a causal link between these two events. First. we could find DAO-5-labeled nucleoli in early *ncl-1* mutant embryos, at a stage when no nucleolus was ever observed in wild type embryos. *Ncl-1* encodes a zinc finger protein that represses transcription by RNA polymerase I and III [29] and we suggest that the earlier appearance of nucleoli in *ncl-1* mutants is triggered by derepression of RNA polymerase I. Also consistent with the existence of a link between nucleologenesis and transcription is our observation of delayed nucleolar formation in the *C. elegans* germline. Indeed, germ cells remain transcriptionally inactive much longer than do somatic cells during *C. elegans* embryogenesis and it is only after the division of the single P4 germ cell precursor into the Z2 and Z3 primordial germ cells, which is thought to be the time of transcriptional activation, that distinct nucleoli could be detected in germ cells (see Figure 4C, F). Prior to that, the staining pattern of nucleolar markers in germ cells is similar to the one found in somatic blastomeres before the appearance of nucleoli (2- to ∼8-cell stage), i.e. 15–25 small foci dispersed throughout the nucleoplasm. Similar observations of delayed nucleologenesis and prolonged maintenance of early GFP-Nopp140 pattern were made in living *D. melanogaster* pole (germ) cells [41].

Some observations are not consistent with a strict temporal link between nucleologenesis and the initiation of embryonic transcription. For instance, it has been reported using *in situ* RNA hybridization that, at least for some genes, embryonic transcription begins at the 4-cell stage, i.e. earlier than the 8-cell stage that had been found to be the onset of EGA by autoradiographic detection of tritiated uridine incorporation [42]. In the case of ribosomal RNA, expression was detected as early as the 1-cell stage using probes against sequences that are only present in the rRNA precursor [43]. We have repeated these experiments and have indeed detected pre-rRNAs in 2-cell and 4-cell embryos in what clearly appears to be a nucleolar pattern, albeit at very low levels (Figure 3). Although we cannot entirely exclude the possibility that these unprocessed or partially processed rRNAs are of maternal origin, we note however that the signals were only detected in prophase and not in S-phase in the earliest embryos, presumably due to the fact that DNA replication, through the doubling of the number of rDNA genes, may have raised transcription above the detection threshold. Increased transcription of rDNA after S-phase has been reported previously [44]. We surmise that the very weak activity of rDNA in the earliest *C. elegans* embryos is not sufficient to nucleate nucleolar markers. It might be that a certain threshold of rDNA transcription is necessary to initiate nucleologenesis and, in this regard, we note again that nucleologenesis in the *C. elegans* embryo coincides with the massive increase in zygotic transcription (including of rDNA) that occurs around the 6- to 8-cell stage.

Since the *C. elegans* genome contains a single rDNA cluster, located near the end of the right arm of chromosome I [45], the observation of two DAO-5 foci per nucleus indicates that nucleoli form on both rDNA clusters in diploid embryonic nuclei and that these remain separate in early embryogenesis. At around the time of gastrulation (∼25-cell stage), the nucleoli are clearly positioned at the periphery in a majority of nuclei. Peripheral positioning of embryonic nucleoli at the onset of nucleologenesis has also been observed in the *Drosophila* embryo [41]. It should be noted however that some *C. elegans* embryonic nuclei harbor both nucleoli in the interior and yet others have one nucleolus in the interior and the other at the periphery. The origin of this variable phenotype is unknown, but we note that in the intestinal precursors and, to a lesser extent, in the hypodermal ones, the nucleoli fuse as development proceeds and that the resulting single large nucleolus is invariably located in the nuclear interior. In *C. elegans* as in other models, localization of gene loci at the nuclear periphery has been associated with lowered expression [46]. It is thus plausible that movement away from the nuclear periphery is a pre-requisite for the fusion of nucleoli and the increase in rDNA transcription and ribosome output with which it may be associated.

The molecular trigger for nucleologenesis in early development remains to be identified. Although the analogy with postmitotic nucleolar re-assembly presents some limitations, in particular the fact that PNBs do not seem to be involved in early embryos [32], it remains useful. Nopp140, already one of the most phosphorylated proteins in the cell, becomes hyperphosphorylated during mitosis and it has been suggested that partial dephosphorylation causes Nopp140 to re-associate with the re-forming nucleolus [47], [48]. It is therefore tempting to speculate that the changing distribution of DAO-5 during early *C. elegans* embryogenesis is associated with changes in the phosphorylation state of the protein. Our initial characterization of nucleologenesis in the *C. elegans* embryo is a first step towards using this well-characterized model organism to better understand the mechanisms of nucleolar formation. The availability of a large set of well-characterized mutants and the ease with which they can be imaged should help to uncover other molecular players involved in nucleologenesis during early development.

## Supporting Information

Figure S1 **Alignment of** ***C. elegans*** **DAO-5 (short isoform, protein Q564W7 from Expasy) and** ***Homo sapiens*** **NOLC1 (hNopp140).** The identity is 46% and the similarity 55%. The part of the protein that was used to raise the antibody used in this study is boxed in red (last 220 amino acids).
(TIF)

Figure S2 **The DAO-5 antibody does not cross-react with human NOLC1.** Human HeLa cells were immunostained with the monoclonal antibody directed againt *C. elegans* DAO-5. No staining is detected in the nucleolus, labeled here with a fibrillarin antibody (green on the right). Rather, the DAO-5 antibody clearly detects punctate structures reminiscent of desmosomes (arrows and red on the right). The differential interference contrast image is shown on the left. DNA is counterstained with DAPI (blue on the right).
(TIF)

Figure S3 **Immunolocalization of nucleolar markers in whole embryos at or shortly before gastrulation.** A) DAO-5 (in red) in a 26-cell embryo. B) FIB-1 (in red) in a 24-cell embryo. DNA is counterstained with DAPI (blue). The entire embryos were scanned by laser scanning confocal microscopy. Shown for each marker are representative maximal projections of top and bottom parts of the embryo (approximately 10 µm in thickness). Mitotic cells are not labeled (asterisks). One nucleus (arrows, that of the germ cell precursor, see text) fails to show the typical nucleolar staining observed in surrounding cells. Images were scanned at the same magnification (pixel size of 95 nm×95 nm). Nuclei appear somewhat smaller after immunostaining for FIB-1 due to slight shrinking during acetone fixation (for DAO-5, samples were fixed with formaldehyde in 1X PBS). Bar: 5 µm.
(TIF)

Figure S4 **A substantial pool of DAO-5 is found in the nucleoplasm of embryonic nuclei.** The DAO-5 signal on a single representative optical section (here from a 45-cell embryo, thickness of 2 µm) was segmented and the pixel number and mean pixel intensity was measured for each segment. The source 16-bit image is shown in A. The mean background pixel intensity was also measured in an area of similar size outside of the nucleus (light blue in the segmented image shown in B). The mean background value is 1306. The signal-to-noise ratio is 2.2 in the nucleoplasm (green) and 13.2 and 14.9 in the two nucleoli (red). The sum total intensity of the DAO-5 signal in the nucleoplasm represents 48% of the total signal intensity measured in the nucleus.
(TIF)

Figure S5 **Co-localization of DAO-5 and FIB-1.** A, D) Single optical sections (thickness of 800 nm) of the DAO-5 signal (red) in nuclei of 2-cell embryos. B, E) FIB-1 signal (green) on the same section. C, F) Overlay of the DAO-5 and FIB-1 signals. The vast majority of signals co-localize, but occasional foci are labeled by only one of the nucleolar markers (arrows in D and E). G) DAO-5 staining (red) in a 3 µm slice through part of a 28-cell embryo. H) FIB-1 staining in the same slice. I) Overlay of the DAO-5 and FIB-1 signals, which co-localize completely. Note that not all nuclei are complete in this rendering of part of a 28-cell embryo and that, as a consequence, not every nucleus displays 2 nucleoli. Bars: 5 µm.
(TIF)

Figure S6 **Electron-lucid zones can be easily identified in** ***C. elegans*** **nucleoli after chemical fixation.** A) Nucleus of a ∼10-cell embryo. The nucleolus appears as a clearly distinct spherical electron dense structure. An electron-lucid zone is indicated by an arrow. A denser region surrounding the other electron-lucid zone is indicated by an arrowhead. This structure is reminiscent of the dense fibrillar component. B) Intestinal nucleus from a L1 larva. Two putative fibrillar centers are clearly seen. C) Higher magnification of the region boxed in B. Arrow points to one of the putative fibrillar center. nu, nucleus; cy, cytoplasm. Bars: A, 500 nm; B, 1000 nm; C, 200 nm.
(TIF)

Method S1 **Ultrastructural analysis after chemical fixation of C. elegans samples.** This procedure was adapted from Vancoppenolle *et al.* [49]. Briefly, young gravid worms were dissolved in bleaching solution (0.5 M NaOH/0.8% sodium hypochlorite) for 5–8 minutes to release embryos. After washing, embryos were treated with 3.4% chitinase/1% chymotrypsin in Egg Buffer (118 mM NaCl, 48 mM KCl, 2 mM CaCl_2_, 2 mM MgCl_2_ and 25 mM Hepes pH 7.3) to remove the eggshell. Samples (total volume of 200 µl) were digested until the shape of the embryos went from oval to round (approx. 12–14 minutes). After addition of 200 µl of L15 medium containing 15% fetal bovine serum, the embryos were pipetted up and down using a fine capillary in order to mechanically remove the eggshell/vitelline membrane. Embryos were then washed in Egg Buffer, resuspended in 100 µl of Egg Buffer, and fixed for 30 minutes at room temperature in Karnovsky’s fixative (2% formaldehyde/2.5% glutaraldehyde in 0.2 M Na-Cacodylate Buffer pH 7.2). After washes in PBS, embryos were included in small agarose blocks, dehydrated, embedded in LR White resin (EMS Inc., USA), and heat-polymerized. Ultrathin sections (60 nm) were deposited on formvar/carbon-coated grids and counterstained with 4% uranyl acetate for 10 minutes and lead citrate for 4 minutes. Observation was carried out on a Zeiss 900 electron microscope.
(DOC)
