# Supplementary figures and images for: Nucleologenesis in the Caenorhabditis elegans Embryo
Source: PLoS One. 2012 Jul 2;7(7):e40290. doi: 10.1371/journal.pone.0040290 (PMC3388055; doi:10.1371/journal.pone.0040290)

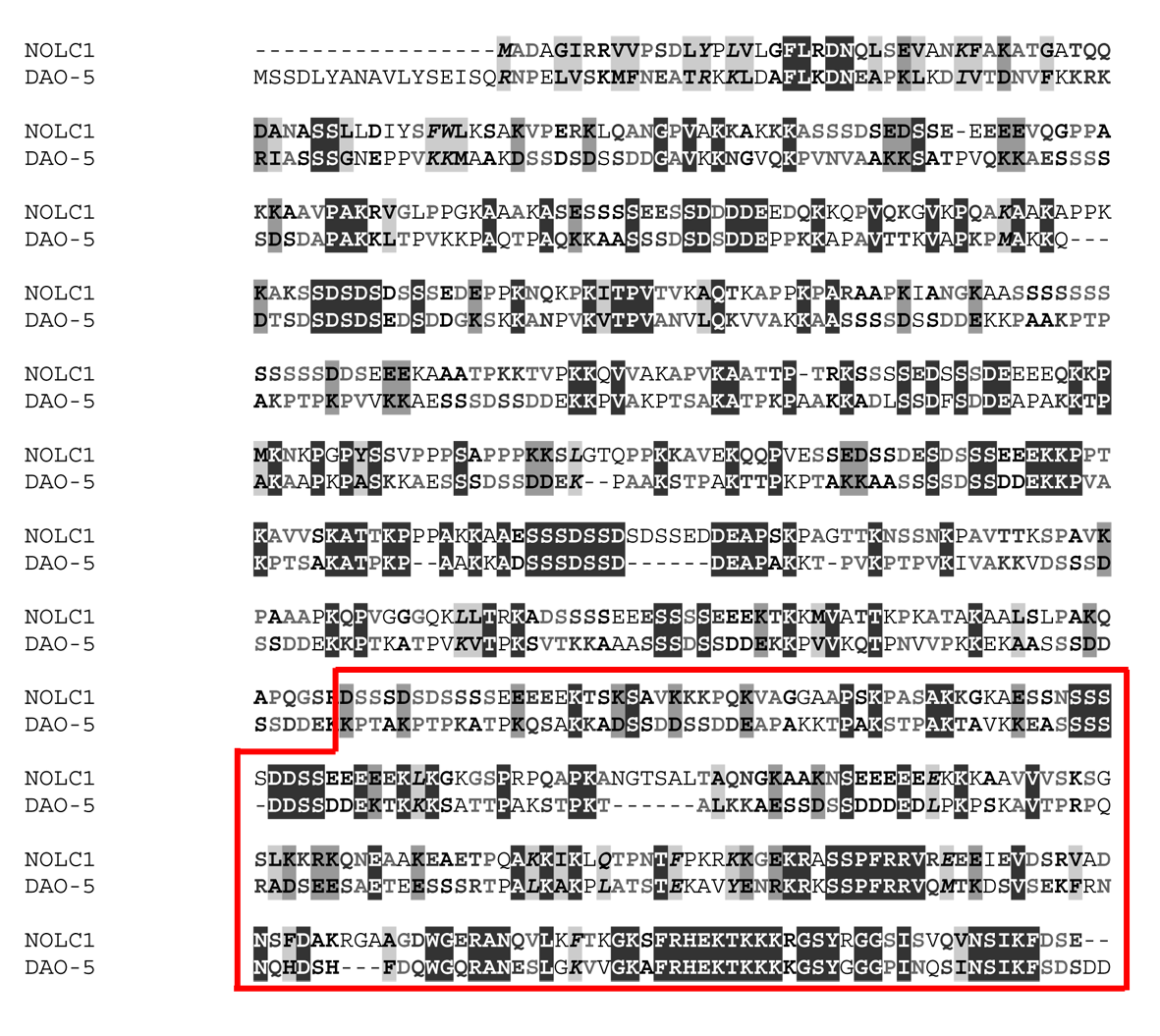

Supplement: Figure S1 — Alignment of C. elegans DAO-5 (short isoform, protein Q564W7 from Expasy) and Homo sapiens NOLC1 (hNopp140). The identity is 46% and the similarity 55%. The part of the protein that was used to raise the antibody used in this study is boxed in red (last 220 amino acids). (TIF) [file pone.0040290.s001.tif]

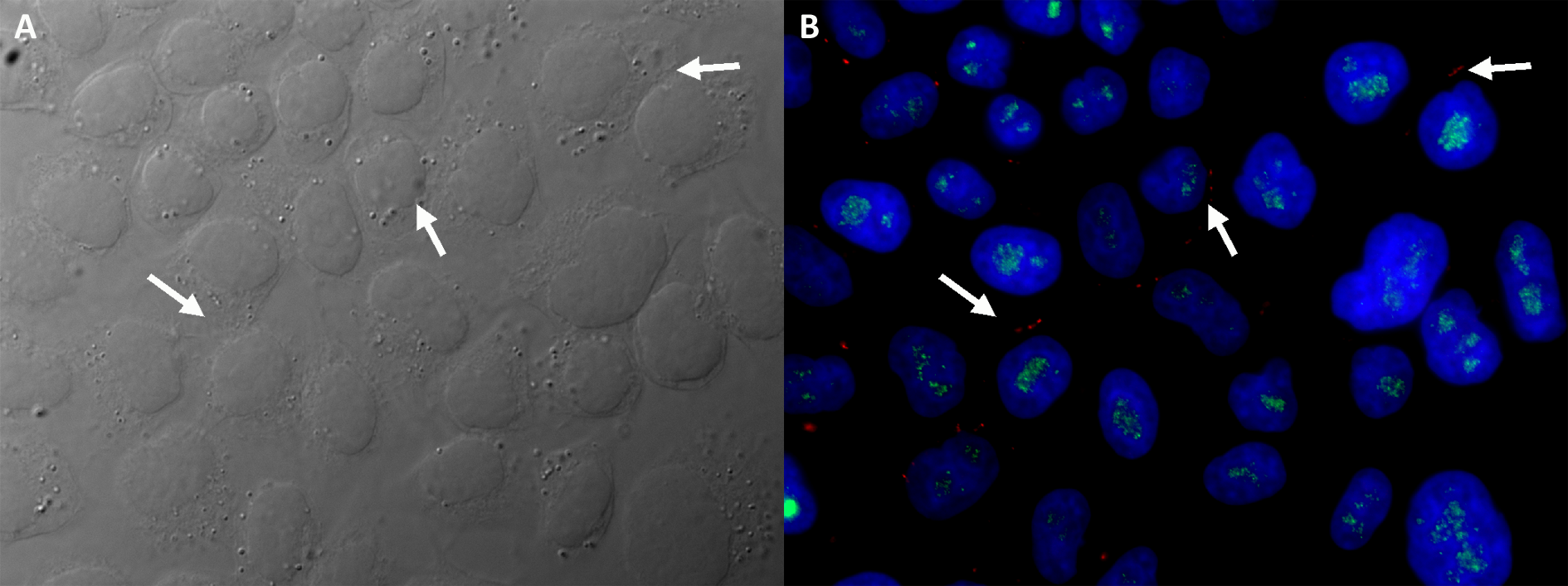

Supplement: Figure S2 — The DAO-5 antibody does not cross-react with human NOLC1. Human HeLa cells were immunostained with the monoclonal antibody directed againt C. elegans DAO-5. No staining is detected in the nucleolus, labeled here with a fibrillarin antibody (green on the right). Rather, the DAO-5 antibody clearly detects punctate structures reminiscent of desmosomes (arrows and red on the right). The differential interference contrast image is shown on the left. DNA is counterstained with DAPI (blue on the right). (TIF) [file pone.0040290.s002.tif]

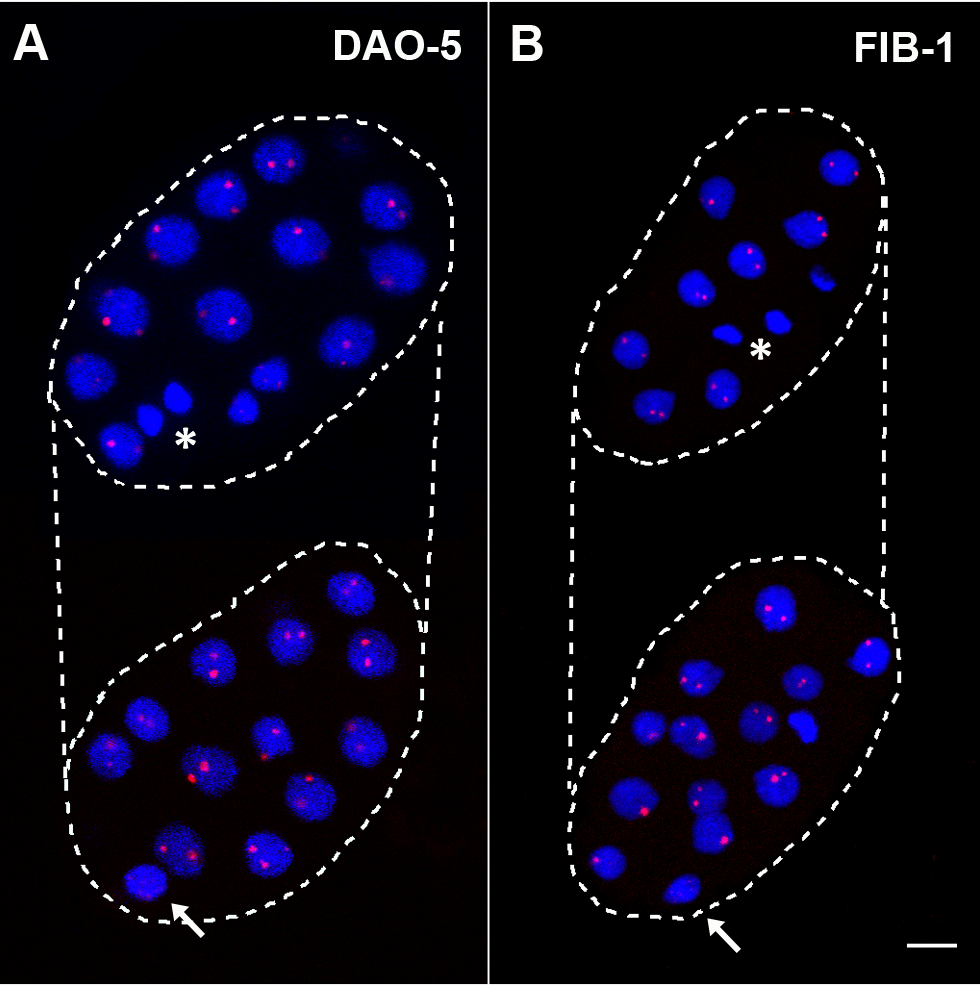

Supplement: Figure S3 — Immunolocalization of nucleolar markers in whole embryos at or shortly before gastrulation. A) DAO-5 (in red) in a 26-cell embryo. B) FIB-1 (in red) in a 24-cell embryo. DNA is counterstained with DAPI (blue). The entire embryos were scanned by laser scanning confocal microscopy. Shown for each marker are representative maximal projections of top and bottom parts of the embryo (approximately 10 µm in thickness). Mitotic cells are not labeled (asterisks). One nucleus (arrows, that of the germ cell precursor, see text) fails to show the typical nucleolar staining observed in surrounding cells. Images were scanned at the same magnification (pixel size of 95 nm×95 nm). Nuclei appear somewhat smaller after immunostaining for FIB-1 due to slight shrinking during acetone fixation (for DAO-5, samples were fixed with formaldehyde in 1X PBS). Bar: 5 µm. (TIF) [file pone.0040290.s003.tif]

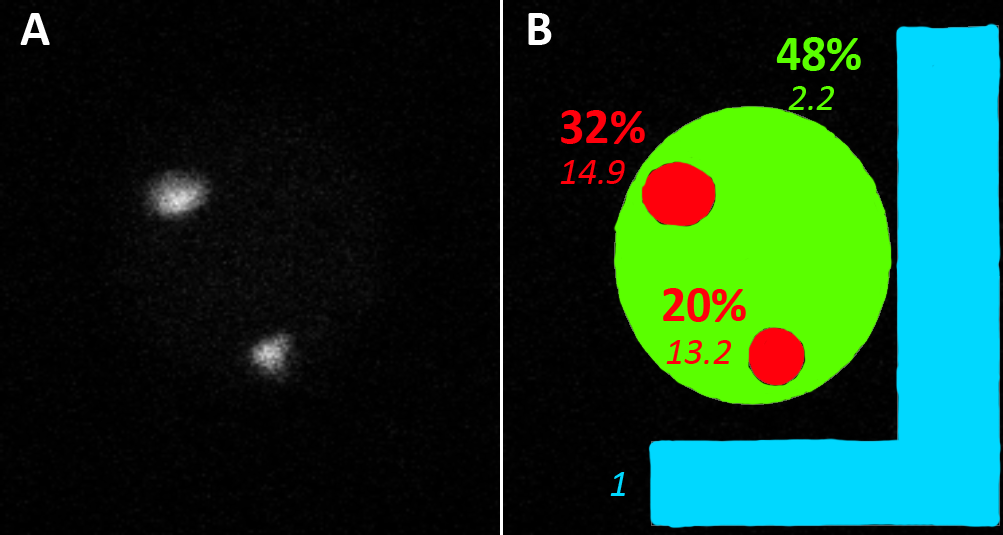

Supplement: Figure S4 — A substantial pool of DAO-5 is found in the nucleoplasm of embryonic nuclei. The DAO-5 signal on a single representative optical section (here from a 45-cell embryo, thickness of 2 µm) was segmented and the pixel number and mean pixel intensity was measured for each segment. The source 16-bit image is shown in A. The mean background pixel intensity was also measured in an area of similar size outside of the nucleus (light blue in the segmented image shown in B). The mean background value is 1306. The signal-to-noise ratio is 2.2 in the nucleoplasm (green) and 13.2 and 14.9 in the two nucleoli (red). The sum total intensity of the DAO-5 signal in the nucleoplasm represents 48% of the total signal intensity measured in the nucleus. (TIF) [file pone.0040290.s004.tif]

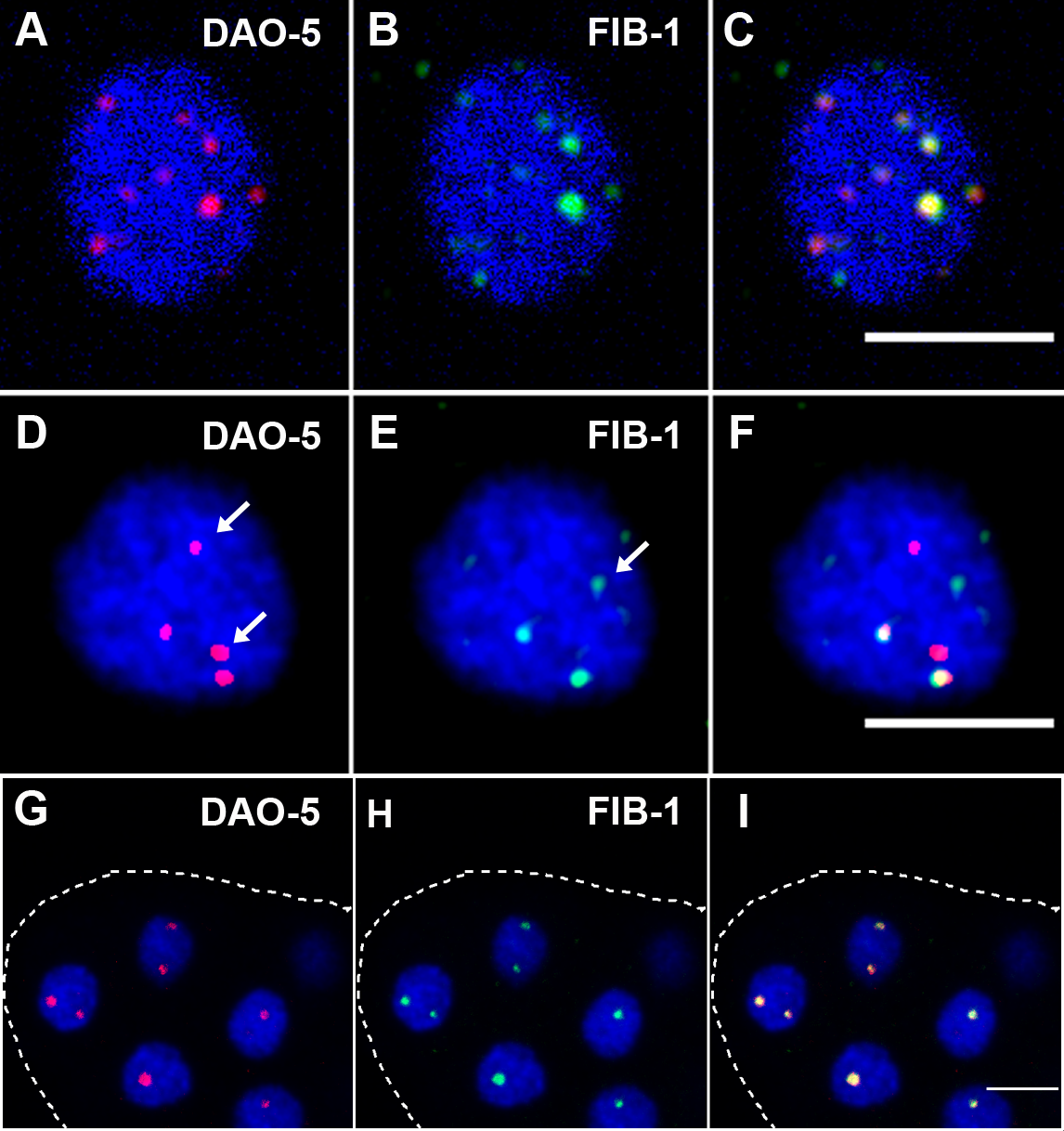

Supplement: Figure S5 — Co-localization of DAO-5 and FIB-1. A, D) Single optical sections (thickness of 800 nm) of the DAO-5 signal (red) in nuclei of 2-cell embryos. B, E) FIB-1 signal (green) on the same section. C, F) Overlay of the DAO-5 and FIB-1 signals. The vast majority of signals co-localize, but occasional foci are labeled by only one of the nucleolar markers (arrows in D and E). G) DAO-5 staining (red) in a 3 µm slice through part of a 28-cell embryo. H) FIB-1 staining in the same slice. I) Overlay of the DAO-5 and FIB-1 signals, which co-localize completely. Note that not all nuclei are complete in this rendering of part of a 28-cell embryo and that, as a consequence, not every nucleus displays 2 nucleoli. Bars: 5 µm. (TIF) [file pone.0040290.s005.tif]

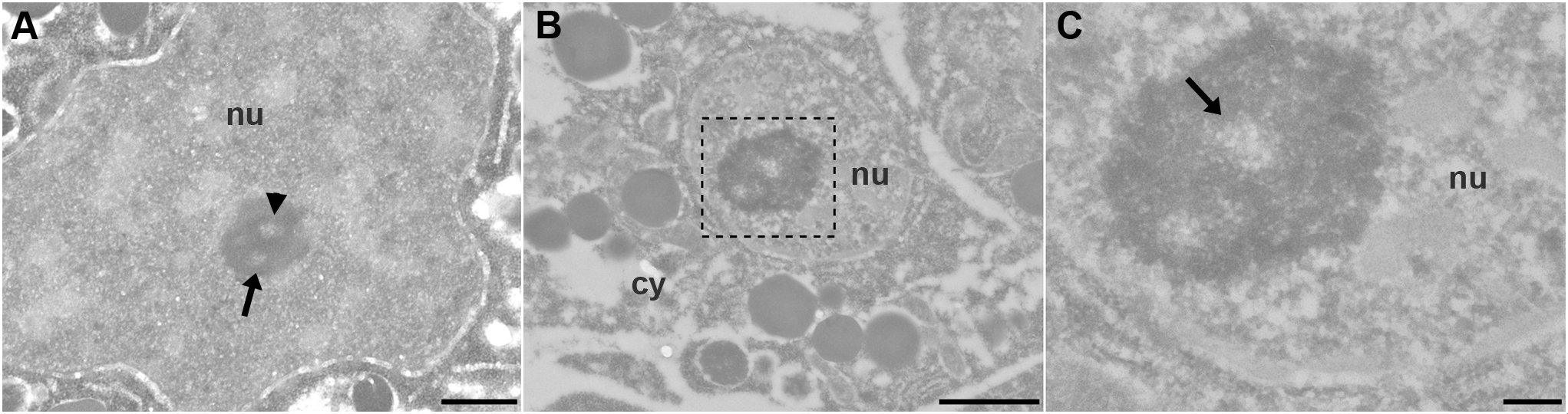

Supplement: Figure S6 — Electron-lucid zones can be easily identified in C. elegans nucleoli after chemical fixation. A) Nucleus of a ∼10-cell embryo. The nucleolus appears as a clearly distinct spherical electron dense structure. An electron-lucid zone is indicated by an arrow. A denser region surrounding the other electron-lucid zone is indicated by an arrowhead. This structure is reminiscent of the dense fibrillar component. B) Intestinal nucleus from a L1 larva. Two putative fibrillar centers are clearly seen. C) Higher magnification of the region boxed in B. Arrow points to one of the putative fibrillar center. nu, nucleus; cy, cytoplasm. Bars: A, 500 nm; B, 1000 nm; C, 200 nm. (TIF) [file pone.0040290.s006.tif]
