# Supplementary material for: Nucleologenesis in the Caenorhabditis elegans Embryo
Source: PLoS One. 2012 Jul 2;7(7):e40290. doi: 10.1371/journal.pone.0040290 (PMC3388055; doi:10.1371/journal.pone.0040290)
Supplement: Method S1 — Ultrastructural analysis after chemical fixation of C. elegans samples. This procedure was adapted from Vancoppenolle et al. [49]. Briefly, young gravid worms were dissolved in bleaching solution (0.5 M NaOH/0.8% sodium hypochlorite) for 5–8 minutes to release embryos. After washing, embryos were treated with 3.4% chitinase/1% chymotrypsin in Egg Buffer (118 mM NaCl, 48 mM KCl, 2 mM CaCl2, 2 mM MgCl2 and 25 mM Hepes pH 7.3) to remove the eggshell. Samples (total volume of 200 µl) were digested until the shape of the embryos went from oval to round (approx. 12–14 minutes). After addition of 200 µl of L15 medium containing 15% fetal bovine serum, the embryos were pipetted up and down using a fine capillary in order to mechanically remove the eggshell/vitelline membrane. Embryos were then washed in Egg Buffer, resuspended in 100 µl of Egg Buffer, and fixed for 30 minutes at room temperature in Karnovsky’s fixative (2% formaldehyde/2.5% glutaraldehyde in 0.2 M Na-Cacodylate Buffer pH 7.2). After washes in PBS, embryos were included in small agarose blocks, dehydrated, embedded in LR White resin (EMS Inc., USA), and heat-polymerized. Ultrathin sections (60 nm) were deposited on formvar/carbon-coated grids and counterstained with 4% uranyl acetate for 10 minutes and lead citrate for 4 minutes. Observation was carried out on a Zeiss 900 electron microscope. (DOC) [file pone.0040290.s007.doc]

**SUPPLEMENTARY METHODS**

*Ultrastructural analysis after chemical fixation of C. elegans samples*

This procedure was adapted from Vancoppenolle *et al.*[1]. Briefly, young gravid worms were dissolved in bleaching solution (0.5M NaOH/0.8% sodium hypochlorite) for 5-8 minutes to release embryos. After washing, embryos were treated with 3.4% chitinase/1% chymotrypsin in Egg Buffer (118mM NaCl, 48mM KCl, 2mM CaCl_2_, 2mM MgCl_2_ and 25mM Hepes pH 7.3) to remove the eggshell. Samples (total volume of 200μl) were digested until the shape of the embryos went from oval to round (approx. 12-14 minutes). After addition of 200μl of L15 medium containing 15% fetal bovine serum, the embryos were pipetted up and down using a fine capillary in order to mechanically remove the eggshell/vitelline membrane. Embryos were then washed in Egg Buffer, resuspended in 100μl of Egg Buffer, and fixed for 30 minutes at room temperature in Karnovsky‘s fixative (2% formaldehyde/2.5% glutaraldehyde in 0.2M Na-Cacodylate Buffer pH 7.2). After washes in PBS, embryos were included in small agarose blocks, dehydrated, embedded in LR White resin (EMS Inc., USA), and heat-polymerized. Ultrathin sections (60nm) were deposited on formvar/carbon-coated grids and counterstained with 4% uranyl acetate for 10 minutes and lead citrate for 4 minutes. Observation was carried out on a Zeiss 900 electron microscope.

[1] Vancoppenolle B, Claeys M, Borgonie G, Tytgat T, Coomans A (2000) Evaluation of fixation methods for ultrastructural study of Caenorhabditis elegans embryos. Microsc Res Tech 49:212-216.
